# Enablers and barriers to implementing an interdisciplinary experiential learning program for university students in a Canadian rehabilitation centre

**DOI:** 10.3389/fresc.2024.1336559

**Published:** 2024-06-03

**Authors:** Jordan Eggiman-Ketter, Benjamin Derrough, Dalton L. Wolfe, Janelle Unger

**Affiliations:** ^1^ Gray Centre for Mobility and Activity, Parkwood Institute, St. Joseph’s Health Care, London, ON, Canada; ^2^ Faculty of Health Sciences, Western University, London, ON, Canada

**Keywords:** interdisciplinary, experiential learning, rehabilitation, program development, implementation

## Abstract

**Objective:**

This qualitative study aims to identify a comprehensive set of enablers and barriers to implementing an interdisciplinary experiential learning program for university students at a Canadian rehabilitation centre.

**Methods:**

A researcher conducted one-on-one semi-structured interviews with individuals from four key stakeholder groups (i.e., rehabilitation centre leadership, clinicians, university clinical coordinators, and health and rehabilitation students). Interviews and data analysis followed the Theoretical Domains Framework (TDF), which is designed to identify possible cognitive, affective, social, and environmental influences on program implementation. Interviews were transcribed verbatim, and two researchers coded data independently to identify the major themes of enablers and barriers to implementing an interdisciplinary experiential learning approach to rehabilitation care.

**Results:**

From a total of 12 interviews, domains of the TDF were identified to represent overarching themes, which were (1) enablers (i.e., reinforcement, beliefs and consequences, optimism, professional identity, knowledge, and skills), (2) barriers (i.e., environment/resources and beliefs and capabilities), and (3) program development (i.e., goals and evaluation that was not previously a TDF domain). A list of recommendations for implementing an interdisciplinary experiential learning program was created that represented qualitative data from each stakeholder group.

**Conclusion:**

This study provides insight into the potential enablers and barriers to developing an interdisciplinary experiential learning program for university students within rehabilitation centres. This type of program could enhance educational curriculums, student and clinical experiences, and patient outcomes. In this study, the findings inform recommendations for developing an interdisciplinary program in teaching hospitals and explore their potential impact. Future research and pilot studies must be conducted to fully understand the effects of implementing an interdisciplinary experiential learning approach within rehabilitation centres.

## 1 Introduction

Teamwork is a key component in rehabilitation centres and as having several disciplines collaboratively working together provides optimal patient care, successful interdisciplinary care is crucial to employ. There are various professions (e.g., physicians, nurses, occupational therapists, physiotherapists) that must work together, and interdisciplinary care can foster better communication and a collaborative work environment (1, 2). Interdisciplinary care differs from other models as team members are more interactive and interconnected rather than working in parallel (3). By having more individuals work collaboratively and provide different perspectives, interdisciplinary teamwork has been shown to improve clinical outcomes and enhance patient care (2, 3).

Alongside interdisciplinary care, experiential learning is another concept that would be beneficial to embed within rehabilitation practices. In this context, experiential learning is a way to introduce students to a more practical and hands-on experience and be able to engage in a clinical setting that is different from the typical class setting (4). Embedding both together and employing an interdisciplinary experiential learning approach will be especially beneficial to involving students from various backgrounds to assist clinical professionals and enhance rehabilitation care. Previous research shows that students who are engaged in both interprofessional learning courses and hands-on experience in a clinical setting have better outcomes in terms of knowledge, confidence, attitudes, and preparation (5, 6). From a student perspective, there are several benefits to engaging in experiential learning opportunities as they can work with other individuals and professionals who differ in background and knowledge (7). There have been programs across Canada that have provided experiential learning opportunities to students and found positive effects (8). Experiential learning is a key component of training for rehabilitation students across Canada; however, currently, there is often no focus on integrating an interdisciplinary approach to the program structure. To combat this limitation, a new interdisciplinary experiential learning program is being developed to ensure the integration of students from various disciplines.

Students gain a deeper appreciation for working in an interdisciplinary manner when exposed to that environment early in their education, which can help inform their future decisions (9). Interdisciplinary experiential learning allows students to appreciate the clinical perspectives of various healthcare professionals/disciplines and the impact that these professionals can have on patients and their families (3, 10). A pilot study conducted by Pechak and colleagues found that rehabilitation students [i.e., occupational therapist (OT), physiotherapist (PT), and speech and language pathologist (SLP)] felt the interdisciplinary course afforded them the opportunity for self-discovery, enhanced collaboration and satisfaction, and a chance to explore outside their comfort zone (11).

Previous research has explored various perspectives regarding interdisciplinary experiential learning from the student and supervisor/advisor perspectives (7, 12); however, these programs involve more than just students and supervisors as the opinions of hospital administrators and university coordinators should also be taken into account to optimally implement an interdisciplinary experiential learning program. One way to obtain this information is by using a framework that focuses on both implementation science and behaviour change from multiple perspectives. The Theoretical Domains Framework (TDF) focuses on those two aspects and can be used in several disciplines to help implement various interventions within healthcare, clinical practices, research, and more (13). For example, one previous study used the TDF to identify teachers' regarded barriers and facilitators to a mandated physical activity policy within a Canadian elementary school (14). This work found that using the TDF aided in understanding and improving future interventions and behaviour change techniques to help with implementation. Since developing and maintaining an interdisciplinary experiential learning program involves and relies on several fields and personnel working together (e.g., healthcare professionals, leaders, and administrators), examining behaviour change is important to successful implementation (15). The TDF combines both psychological and organizational theories and evidence-based recommendations to target specific behaviours that will lead to sustainable changes to support the intervention (16, 17).

This study aims to inform program development by interviewing key stakeholders about the implementation of an interdisciplinary experiential learning program informed by the TDF model. Understanding the various enablers and barriers to implementing this program can help spread awareness and support developing successful interdisciplinary experiential learning initiatives for university students in rehabilitation centres.

## 2 Methods

This qualitative study follows a phenomenological approach and was approved by the Western University Health Science Research Ethics Board (HSREB) in accordance with the Declaration of Helsinki. Informed consent was obtained from all participants prior to beginning the interviews.

### 2.1 Participants

For this study, members from four key stakeholder groups were interviewed: rehabilitation centre leaders, clinicians, university clinical coordinators, and students from various health and rehabilitation disciplines. Rehabilitation centre leaders oversee and coordinate the programs at the rehabilitation centre. Clinicians are supervisors of students from different health disciplines (e.g., physical therapists, physicians, and nurses). University clinical coordinators work within the university and are responsible for student organization and ensuring the program fits educational curricula. Students are from several health and rehabilitation areas who have taken part in experiential learning. Purposive sampling was used, where members of the research team identified potential participants.

### 2.2 Materials

The semi-structured interview guide was formatted to be one-on-one with open-ended questions and was developed by the research team with backgrounds in health science, rehabilitation science, and psychology based on the Theoretical Domains Framework (see Table 1). The Theoretical Domains Framework aims to provide guidance on successful implementation and is designed to identify various influences on behaviour (e.g., cognitive, affective, social, and environmental) that could impact program implementation (13). By using a semi-structured interview, the participants were able to share relevant details to the study goal while also providing additional information that they felt was relevant to the study.

**Table 1:** Original and adapted descriptions for the relevant domains used from the theoretical domains framework (13).

TDF domain	Original TDF descriptions	Adapted TDF description
Knowledge	An awareness of the existence of something	Anything that involves gaining more knowledge of interdisciplinary care
Skills	An ability of proficiency acquired through practice	Desirable skills of incoming students
Environment Context and Resources	Any circumstance of a person's situation or environment that discourages or encourages the development of skills and abilities, independence, social competence, and adaptive behaviour	Structural or organizational circumstances that may help or hinder the implementation of an interdisciplinary experiential learning program
Social/Professional Role and Identity	A coherent set of behaviours and displayed personal qualities of an individual in a social or work setting	Aligning values of an interdisciplinary experiential learning program and one's personal or professional values
Beliefs and Capabilities	Acceptance of the truth, reality or validity about an ability, talent or facility that a person can put to constructive use	Ability to engage and be involved in an interdisciplinary experiential learning program
Optimism	The confidence that things will happen for the best or that desired goals will be attained	Confidence in the program's development and/or its values and goals
Goals	Mental representations of outcomes or end states that an individual wants to achieve	Ideas or suggestions made that would help achieve and support the desired outcomes of the program
Beliefs and consequences	Acceptance of the truth, reality, or validity about outcomes of a behaviour in a given situation	Opinions or thoughts on what outcomes could occur from this initiative
Reinforcement	Increasing the probability of a response by arranging a dependent relationship, or contingency, between the response and a given stimulus	Circumstances or individual capabilities that help support/hinder the startup of the program (e.g., motivation and incentives)
Evaluation^a^	NA	Methods for measuring the success and progress of this program

^a^ Not an original domain from the TDF.

### 2.3 Procedures

#### 2.3.1 Data collection

All participants provided informed consent prior to taking part in the interview. Interviews happened either in person (*n* = 2) or through Microsoft Teams (*n* = 10) by a researcher with background knowledge in rehabilitation sciences and psychology (JE-K, undergraduate student). Each interview was audio-recorded and transcribed verbatim.

Using an iterative process, the researcher utilized previously captured knowledge to assist with subsequent interviews and ask additional questions.

#### 2.3.2 Data analysis

Two members of the research team (JE-K, a psychology student, and BD, a kinesiology student) coded the data following coding guidelines based on the Theoretical Domain Framework (TDF; 13). The descriptions of the TDF domains were modified slightly from the original descriptions to fit the scope of this project (see Table 1). The researchers first individually identified the information broadly into categories as being an “enabler” (something that supports the initiative), “barrier” (an obstacle to implementing the initiative), or “neutral” (perceived as important information that is not directly an enabler or barrier). These categories were later grouped into subcategories representing an adapted version of the TDF approach, and each researcher coded data into the TDF subgroups.

The second phase of coding involved collapsing together the separately coded interviews to compare and review the similarities and/or differences in coding that emerged. The codes were classified following the adapted TDF approach, although information could be coded in more than one domain. A list of recommendations for implementing an interdisciplinary experiential learning program was made based on the categories and subcategories. This list was sent to an individual from each stakeholder group so they could review the recommendations and provide any additional comments or suggestions for member checking.

## 3 Results

A total of 12 participants were interviewed with each stakeholder group consisting of 3 participants, and the length of interviews ranged between 30 min and 90 min. The results of this study placed the domains of the TDF into three major themes regarding the implementation of an interdisciplinary experiential learning program within rehabilitation centres. The overarching themes were (1) enablers, (2) barriers, and (3) program development (see Table 2). The TDF domains were categorized as representing a certain theme based on the highest coding proportion they had within that theme (e.g., environment/resources accounted for a greater proportion of barriers than either enablers or neutral).

**Table 2:** Description of stakeholder group, role, and coding ID.

Stakeholder group	Role description	Coding ID
Rehabilitation centre leaders	Oversee the programs at the rehabilitation centre and coordinate them	H01—Rehabilitation Coordinator H02—Physician H08—Nursing Leader
Clinicians	Supervise students from different health disciplines (e.g., physical therapists, physicians, and nurses)	C03—Physiotherapist C10—Occupational Therapist C12—Speech Language Pathologist
University clinical coordinators	Work within the university and are responsible for organizing the programs and ensuring that the program fits both the university and rehabilitation centre's standards	U04—Academic Coordinator U06—Academic Program Manager U11—Experiential Learning Coordinator
Students	From several health and rehabilitation areas and have already had a practicum experience	S05—University Alumni Student (Practicum student) S07—University Alumni Student (Independent study student) S09—University Alumni Student (Practicum student)

Table 2 describes each stakeholder group and participant title/role with their coding ID to help indicate which stakeholder group each quote represents. The various quotes that represent the perspectives of implementing a new program in alignment with the TDF approach are shown in Table 3. The coding coverage for each domain categorized under each of them is represented in Appendix C.

**Table 3:** Participant quotes representing each TDF domain (13).

Quotes		
Theme	TDF domain	Quote
Enabler	Reinforcement	H02: “there's some feasibility- the good news from our side of it, from the planning perspective- is we have almost complete control over the schedules that we put together when students rotate at [rehabilitation centre], so it's easy for us to make those changes from a planning perspective” H01: “showing how it's good for patients, their families, clinicians, and students then that helps with buy-in because it is something that our organization would be supporting so I think that that would help down the line”
	Beliefs and consequences	C10: “I think it is worthwhile to have the interdisciplinary approach because perhaps you're going to have students coming into professions with a more clear understanding of what the profession is about. And I think, I think if it improves patient care, which I know it does, then we should all be for that” U04: “why we want this initiative, it's the student learning and preparation for practice but then that is ultimately feeding back into that patient outcomes so I mean, are we really benefitting the patients and the clients. Yeah, I don't know- students and patients”
	Optimism	U04: “there's interest, there's momentum, there's engagement and those are kind of the key things. So, I think that it is absolutely feasible because it's not just kind of one person driving the initiative, we have a whole team so I would say “yes” for those reasons”
	Professional identity	H08: “[Hospital network] is definitely- that's one of the strategic directions or Mission/Vision values type of thing is to support- to be a partner with an academic center and be in support of student experience and I think doing it in an interdisciplinary fashion would also be like supported”
	Knowledge	U11: “Interdisciplinary experiences, I think can be super valuable because they simulate a lot more of like, what the real world is and probably more of like, like, if they were working at [rehabilitation centre] not as a practicum student but as an employee or researcher” C12: “We’re constantly raising awareness when we have practicum students in the building, raising awareness of our own disciplines. Uh, or raising awareness, uh, within the patients of, uh, things that may help them”
	Skills	H01: “being open minded, willing to participate in development, and offering ideas, working well in a team because typically we have more than one student, so they have to work well with others” S09: “collaboration is kind of the name of the game in interdisciplinary practices” U04: “we want students who are passionate, interested, curious, and so having students apply for such a placement increases that engagement a little bit and that has worked well for multiple initiatives that we’ve run already”
Barrier	Environment/resources	H08: “it's more like the actual feasibility, like the logistics, the how-to, the who is going to be involved, how it's going to impact our staff, like that's where the leaders would come with more of a that that like the cost”
	Beliefs and capabilities	C10: “the barrier is and you'll probably hear this that, oh, it's hard enough just to get an OT placement and asking if they, can we, is there request to clinicians to add another placement request for say a medical resident or social work student to come and observe me and all of us on our team, the social worker, the speech path, the physio sometimes, the assistant, we might all have students at different times, so, we already feel like we're tapped out with having students” C10: “it's just our days are jam-packed with day clients and writing notes and it's all slowed down by having a student because then it's taking- you want to explain things”
Program development	Goals	C03: “helpful to have someone who was trained on the frontline and kind of understood how that worked but then was almost like a liaison between the frontline staff and some of the management pieces” U06: “having the conversation about building these opportunities out is how do we back that down into the curriculum to ensure that interdisciplinary fourth year experiences are really valuable to students and they have the skills and knowledge to really, you know, apply that in fourth year.” U11: “it's like how you package it, and how you talk about it and how you present it to people. It's super important … It is new so like how you roll it out and how you tell the story of it I think is really important”
	Evaluation	H08: “focus group or surveys, maybe having some baseline and then having the students go through the program and then repeating maybe a survey and have some focus groups to kind of share their reflections and maybe some reflective practice throughout the experience”

### 3.1 Theme 1: enablers

Codes were identified as being *enablers* when participants mentioned anything that would support the implementation of an experiential learning program or when the participant had a positive outlook about the factor. The following TDF domains were identified as being *enablers*: reinforcement, beliefs and consequences, optimism, professional identity, knowledge, and skills.

#### 3.1.1 Reinforcement

Reinforcement was represented as anything that was foreseen to help support or hinder the startup and continued participation in the program (e.g., motivation, enthusiasm, and incentives). The focus is on what things can foster a continuous drive to engage and be a part of the program. Overall, there was a description of support from the key stakeholders in terms of initiating an interdisciplinary experiential learning program. All participants recognized the benefits this program could have on learning and patient outcomes within the healthcare environment for students, hospital staff, and patients, which facilitates motivation and incentives to engagement. One student described a practicum course positively saying that it “didn't feel burdensome unlike some of my classes. So yeah. So that was great. It was like a little break like in a weird way. Yeah just such a change of pace from the usual academic” (S09).

Ensuring student programs support patient care is crucial as one hospital leader said “showing how it's good for patients, their families, clinicians, and students then that helps with buy-in because it is something that our organization would be supporting so I think that that would help down the line” (H01).

Not only was there support on a personal level, but from a university organizational perspective, one coordinator said “I think we're, we're ready. We're willing, I think, um, students are looking forward to it. It's timely given sort of the conversations we're having around the undergraduate curriculum. We have experiential learning coordinators, one now focused on partnerships and so I think, you know, there's a lot of moving parts that now align really well with helping support moving this forward” (U06).

#### 3.1.2 Beliefs and consequences

Beliefs and consequences were described as opinions or thoughts on what outcomes could come from this initiative and then extend beyond the participation of the program. This domain focuses on just the acknowledgement and perception of a potential outcome (positive or negative). The participants mentioned more favourable outcomes than negative ones, which highlighted the positive impact on students, clinicians, patients, and the organizations associated with this initiative. Increased workload on supervisors and clinicians was mentioned but many stated that having a strong student can help with assistance and ultimately aid in providing better care to patients.

One clinician noted that, with an interdisciplinary experiential learning program, “the outcomes are just going to be better. Because patients feel heard, supported from all parts of who they are, and when an interdisciplinary team's there” (C12).

All stakeholders recognized the importance that this interdisciplinary program holds for participating students as they can “gain the confidence early on or opportunities to see what [they] like or what [they] don't like or what [their] strengths are- that maybe [they] wouldn’t have an opportunity to do that before I personally think is a really important part of learning and probably the university experience” (H01).

#### 3.1.3 Optimism

Optimism was described as having confidence in the program's development and/or its values and goals. The participants were confident in the program's values, and all of them highly rated the importance of having an interdisciplinary experiential learning program.

Students were especially supportive of this initiative with one saying it “was truly probably the most valuable experience I took away from undergrad. So, I think it would be really important to make sure future students get that same thing” (S09). Clinicians also noted how beneficial this type of program is when students are “physically getting in there and getting your hands on things. So, I think that as an overall learning experience for the students, I think it's kind of unmatched” (C03). Another participant noted that “it's not just feasible, it's probably necessary” (C12), in terms of providing these types of learning opportunities to students in rehabilitation centres.

#### 3.1.4 Professional identity

Professional identity relates to the extent to which implementing an interdisciplinary experiential learning program aligns with one's personal or professional values. All participants agreed that this initiative lines up with their ideals either in a personal/career path manner (e.g., personal beliefs or help with future aspirations) or in a professional/organizational sense (e.g., organizational mission or strategic planning).

Integrating an interdisciplinary experiential learning program incorporates values from both the hospital and university perspectives. One hospital leader noted that a goal is to “promote here at [hospital network], you know, partnership and collaboration, and team approach, and involving a patient and caregiver and that kind of thing. So, I think definitely aligns with what the organization wants to see” (H08). From the university's perspective, “this initiative definitely lines up with some strategic priority, not just within the [School] but broader within the [Faculty]” (U04).

Students also recognized the value of participating in an experiential learning opportunity for their future professional identity, as one explained that “really getting to experience it and observe it all was yeah, so so important, I think for shaping the kind of clinician I want to be down the road and even more broadly, the kind of person I want to be, like my professional identity wise” (S09).

#### 3.1.5 Knowledge

Knowledge was classified as anything that involved an individual, mainly students, gaining more knowledge of what interdisciplinary care is and how to work within an interdisciplinary setting.

One student said that “it can only add to like their experience, learning in the practicum and just give them more of an idea of what they might have to do after school” (S07), and another mentioned how students could take their “knowledge and kind of disseminate it into the general community, in the public to greater inform, you know that every day, lay people about the different interdisciplinary healthcare professions is also kind of a more valuable component of it” (S09).

One hospital leader noted that from the students' perspectives, they can “increase their comfort level, increase their understanding and knowledge, increase their confidence. In you know, how to like, appreciating different disciplines and their roles and how they work and so on and being able to and how they contribute and be able to actually do that on- with mentorship from the clinicians. And appreciating the team approach and following that study” (H08).

Overall, having an interdisciplinary experiential learning program could promote “increased knowledge and understanding of the interdisciplinary team approach of what various team members can contribute. And increased maybe confidence in how the interdisciplinary care can be delivered” (H08).

#### 3.1.6 Skills

Skills were coded whenever participants mentioned what they believed would be ideal or desirable skills for incoming students to have. Most participants deemed collaboration and communication as key assets to have, as well as being able to work professionally and adapt to changing situations. They noted the benefit of students having an interest and bringing enthusiasm with them when working with the clinical team and helping with the care of patients.

Various types of skills were noted to be critical for students to be “adaptable to changing situations or are able to be flexible, because a lot of these things it's hard to predict what exactly they're going to look like so you need a student who is okay with learning on the fly or things changing” (H01), to have “curiosity and initiative” (C12), and to have “super soft skills regardless of what they choose to do. It's like you were working collaboratively, you have to create like think critically and like work in teams, and in like interdisciplinary environments” (U11). Some student stakeholders reflected that students should be “interested or open to participating in this and being motivated to like meet other people through this program” (S07) and be able to “foster, facilitate the development of students who are able to function well in a team, communicate efficiently, professionally, and kind of convey their messages well, accept feedback graciously and have kind of an invested interest in self-improvement and self-reflection” (S09).

### 3.2 Theme 2: barriers

The codes were identified as being *barriers* when participants mentioned anything that would hinder or prevent the implementation of an experiential learning program or when the participant had concerns about the development or impact of this initiative. The following TDF domains were identified as being *barriers*: environment/resources and beliefs and capabilities.

#### 3.2.1 Environment/resources

Environment and resources were depicted as any structural or organizational circumstances that may help or hinder the implementation of an experiential learning program. Most barriers were indicated as being environmental or resource issues. Many participants mentioned that this initiative could be burdensome to students and clinicians, which could counteract the benefits. One student participant recognized that “students are very very busy and same with clinicians” and if the program was voluntary, there “might just have less people showing up because of busy schedules” (S05). Similar concerns by supervisors as one clinician noted that there is the aim of trying “to get a lot of people in to experience but it can't be so much so that it's taxing on the clinician that we’d feel like we’d have to keep track of too many people and space” (C03). Not only the workload of different groups needs to be considered but also the planning and structure of the program as “the logistics and kind of how it would be done would need to be brainstormed and figured out” and how to “engage the stakeholders who would be like involved to help develop it” (H08). Another participant highlighted some key questions that would have to be considered such as, “who facilitates that [program], right? Like if it's an interdisciplinary thing like is that like a staff in one area? Like what does that workload look like?” (U11).

#### 3.2.2 Beliefs and capabilities

Beliefs and capabilities were coded as the perceived ability to engage and be involved in this program. This was a foreseen barrier to the implementation of this program and many participants had concerns about the capacity that clinicians, students, and faculty members would have to implement and sustain this program. Concerns were mostly identified from the organizational/logistical side of implementing a new program into a hospital or ensuring that clinicians are not being overtaxed by working with students. There are a lot of considerations that need to be accounted for and working between two major organizations (i.e., hospital and university) can be a “large undertaking … to try to like, bring everyone together because there's just so many different stakeholders” (U11).

However, for students, many felt that participating in this program was achievable, especially if it is embedded into the curriculum. One student who was part of a practicum course said, “from the student perspective, I think if it's incorporated into the curriculum, then it would be easy. The minute you sort of require additional work I think that's where you might lose some of that buy-in” (S05).

### 3.3 Theme 3: program development

The codes were identified as *program development* when anything specific to the creation and planning of the program was brought up. Several participants mentioned various things that should happen before and during the program that would increase its sustainability and efficiency. This theme differs from the others as it focuses on the planning process and structuring of the new program rather than its intended outcomes. The only TDF domain that was classified in *program development* was goals. An additional subcategory was created, evaluation, as it was identified as an important factor to address when implementing any type of programming and to measure goal achievement.

#### 3.3.1 Goals

Goals were ideas or suggestions made that would help achieve and support the desired outcomes of the program. Several participants noted some goals they would like to see happen when implementing a new program. This initiative involves a lot of people and coordination so having “someone who is trained and knows the frontline area but can also work to bring up kind of the important issues to management and work to help them and come up with strategies to implement” (C03) is a key aspect to consider. Engaging students early on in their academic career was also noted as something that could be beneficial as it helps in “providing some background as to what might be expected of them and having students apply I think is a great thing and really speaks to including students that would be a good fit for some of the things, especially in the early stages” (U04).

#### 3.3.2 Evaluation

Evaluation was not originally in the TDF as a domain but was added to this study to further understand the appropriate methods to measure the success and progress of this program. One hospital leader noted some things to consider when measuring the success of the program which include, “are our patients satisfied, we've improved the care that they received, either by decreasing the waitlist, increasing our volumes, while not like overtaxing the staff, and improving the experience of students” (H01). The participants also mentioned that having feedback from the different groups involved (i.e., students, preceptors/supervisors, and patients) is important, particularly when it is reciprocal. One student mentioned that it is “great for feedback to work in both ways so that we can also give some feedback to the, like our preceptors” (09). The participants mentioned some evaluative methods which could include focus groups, surveys, online discussions, or reflections as ways to provide feedback back to students or preceptors and to help inform how the program is running.

## 4 Discussion

The findings of this study inform the implementation of an experiential learning program with an interdisciplinary approach. The results contribute to understanding what supports, encourages, or hinders the development and implementation of a new program at a rehabilitation centre and what is needed to appropriately roll out this initiative. Although this study focused on the barriers and enablers, many of the participant's comments addressed considerations for the development of the program. Most findings were positive, and this type of program is believed to have a beneficial impact on several groups, including students, clinicians, and patients. Having an interdisciplinary experiential learning program aligns with both the hospital and the university's strategic priorities and therefore creates a positive impact for the involved organizations. However, there were some logistical concerns about implementing a new experiential learning program within an existing rehabilitation centre.

To address these concerns, a set of recommendations were made to help with the development and implementation of an interdisciplinary experiential learning program at rehabilitation centres (see Table 4). It should be noted that this study took place just after COVID-19 restrictions, which had lasted around two years, started to be lifted. Many participants mentioned the effects that COVID-19 had on the rehabilitation centre, educational experiences, and/or their personal lives. Most feedback did not pertain to the specific effects of COVID-19, but issues such as safety concerns and spacing issues were brought up with the pandemic in mind.

**Table 4:** List of recommendations for implementing an interdisciplinary experiential learning program.

Recommendations		
Theme	TDF domain	Recommendations
Enabler	Reinforcement	a. Discuss planning of student placements well in advance b. Share testimonials of successes from the program
	Beliefs and consequences	Encourage interdisciplinary approaches and understanding of various health professions and disciplines to support students in a clinical and academic setting
	Optimism	Determine an optimal time for rehabilitation centres and educational institutions to start planning and implementing an experiential learning program for students
	Professional identity	Incorporate institutional priorities/values (e.g., strategic planning)
	Knowledge	a. Encourage students to reflect on their future goals/desires and how participating in an interdisciplinary program can support those goals b. Spread awareness and understanding to clinicians on how supporting learners can aid patient care and their own experiences/knowledge
	Skills	Identify students who are highly interested and professional, and can collaborate and communicate effectively
Barrier	Environment/resources	Discuss logistical barriers (e.g., cost, processes, facilities) and develop a strategy on how to address foreseeable issues (e.g., staff impact, structure)
	Beliefs and capabilities	a. Focus on reducing clinician burden, or perception of burden b. Reflect on how to best transition students to a healthcare setting
Program development	Goals	a. Embed into current curriculum early on in undergraduate career (1st or 2nd year) to help inform their choices in upper years and post-graduation b. b. Have someone who can champion and oversee the program (e.g., manage between the hospital and university or between frontline and leadership teams) c. Consider the promotion of the program and how to properly inform/engage students (especially if it is a new program)
	Evaluation	Utilize feedback and reflections from students and preceptors/supervisors involved in the program

### 4.1 Enablers

The results indicated that there are several benefits to incorporating a collaborative and engaging program in hospitals. These include enhancing student involvement and knowledge in clinical healthcare settings which aligns with previous research on the benefits of hands-on experiences (5).

Recommendations were made based on the *reinforcement* codes, which were to plan the structure and process of placements well ahead of students being onboarded and to spread awareness of the successes achieved through the program after implementation. Doing so will help with organization, provide a smooth transition, and showcase the benefits of implementing an interdisciplinary experiential learning program to various groups (e.g., students, clinicians, patients, and organizations). This work will facilitate the sustainability of the program and re-involvement of participating parties when new programming occurs. To produce enabling *beliefs and consequences*, encouraging proper understanding of interdisciplinary settings and the various disciplines involved in health care can further support students' engagement in their learning. These findings align with previous research where students were reported to enjoy working collaboratively as it helps with understanding, problem-solving, and providing better patient care (5, 18). If students can optimize their contributions, patient care can be positively influenced and allow for a holistic approach (19). *Optimism* in this type of program was achieved when the timing was optimal for all parties involved. If the timing and management work for all organizations, confidence in the success of this program increases drastically, especially when there are pre-existing programs associated with the hospital/educational institution. Ensuring that organizational values and mission align with the proposed program and maintaining a *professional identity* by incorporating institutional priorities and strategic planning is crucial. Aligning the program's values with the associated organizations can enforce the significance of the program as well as produce a reciprocal and mutual benefit to all parties involved (20, 21). The factors that would increase *knowledge* in participating individuals include having students reflect on how engaging in an interdisciplinary experiential learning program can support their goals. If members are motivated by the initiative, learning will result in an increased understanding of interdisciplinary healthcare settings. Another recommendation was to spread awareness of the benefits of employing an experiential learning program to clinicians and/or supervisors in particular, so they can help educate incoming students and work towards enhancing patient care. These findings are similar to the study by Van Wyk et al. (19), where the authors found simulating an interdisciplinary setting can encourage students to work more effectively within multiple disciplines. The participants mentioned several *skills* they would like students to have and how identifying students who are strong in these skills (e.g., teamwork, collaboration, motivation, communication, and professionalism) will support the viability of the program.

### 4.2 Barriers

The findings demonstrate that there are logistical concerns that need to be addressed before implementing a new program. This would include *environmental/resource* barriers, which could be addressed by having discussions about costs, having access to facilities (e.g., space, capacity, and safety), and sorting out appropriate processes (e.g., onboarding of students, dealing with challenging students, and facilitators). With new programming, there will be an extra cost to taking on a new initiative and embedding it into practices (5). These costs could be monetary but also from other resources such as time, energy, and coordination. It is important to have strategies in place on how to address these issues before they arise. Along with costs, organizing the use of facilities (rooms being used, spacing within rooms, etc.) needs to be decided on as there will be additional people within the rehabilitation centre. For both issues, determining and regulating the processes are key to developing sustainable programming. Additionally, clinicians who would be supervising the students need to feel supported and confident in who is assisting them and not overly burdened. Additional programming is a large commitment and takes many resources from various areas such as staff members, students, and organizations (22, 23), which is why there needs to be a focus on reducing this potential burden. The perceived *beliefs and capabilities* of involved members need to have minimal risk to decrease the reservations that clinicians have about supervising students. There also needs to be uptake in students who are motivated to take part in this kind of initiative, which means planning out and communicating with students about how they are going to be supported in their transition into a healthcare setting.

### 4.3 Program development

The potential outcomes of a program are another crucial consideration in the development of a new program and how it is structured. With multiple disciplines involved, the way the program is rolled out and how everyone will be coordinated will affect how successful it is in delivering enriched education and optimal patient care (5). There were several *goals* that participants had mentioned they would like to see achieved prior to or during the implementation of this program. One, the program would be embedded within the student curriculum and would occur early on in either the first or second year of training. As Salvatori et al. (24) noted, coordinating student timetables is an obstacle, and uptake is difficult when students perceive it as additional work on top of their academics. This aligns with what university clinical coordinators mentioned in this study, which is to embed the program within student academics and introduce it in the earlier academic years. This will help students make more informed career choices and have a better idea of future directions in their later years of training. A second goal is to have someone champion or manage/oversee the program to help organize and orient students/supervisors. Lastly, the topic of how to properly promote the program and entice students to get involved in this initiative was mentioned. Since this would be a new program being offered, uptake may be difficult to achieve in the beginning. Similarly, Copley et al. (23) highlighted the importance of promoting interprofessional education and how framing can help support implementation within clinical practices. Offering an experiential learning program to students can have significant benefits to the participating parties, but if the initiative is not packaged engagingly, enrollment and retention will be difficult to maintain. Another recommendation was regarding the *evaluation* of the program and what potential methods could be used. This sub-theme was not originally part of the TDF but was an additional consideration as evaluating programs is important to the implementation and sustainability of a program (25). Receiving feedback from a variety of perspectives such as hospital leaders, university clinical coordinators, students, and clinicians is an important step to take to ensure that the program is supporting all parties as well as providing beneficial learning and care outcomes. This is especially important since different stakeholders will be interested in evaluating different components of the program and analyzing various metrics. This finding aligns with previous research that notes the importance of allowing reflection to occur for experiential learning opportunities to help improve the structure of the experience (22).

### 4.4 Limitations

A limitation of this study is the reduced generalizability of the findings to other regions due to the sampling methods employed. The data captured stems from the participants who were all currently working at the same rehabilitation centre or were affiliated with the same university institution. While there was representation from different disciplines (i.e., *clinicians*—speech and language pathologists, occupational therapists, and physical therapists; *rehabilitation centre leaders*—spinal cord injury rehabilitation navigator, physiatry, and nursing), it is acknowledged that not all voices within a rehabilitation centre were included in this study.

### 4.5 Future directions

This study provides insight into the development of an interdisciplinary experiential learning program at rehabilitation centres. Although this study was based in Ontario, the information and knowledge provided can be generalized to other Canadian institutions and organizations. However, future research should focus on expanding outside of the Canadian context as well as implementing pilot interdisciplinary experiential learning programs. Further research in this area could result in a change in academic curricula to include more interdisciplinary experiential learning for students. Offering more opportunities to gain practical skills and knowledge will facilitate the development of healthcare providers and leaders which will in turn enhance patient outcomes in rehabilitation centres.

## 5 Conclusion

In conclusion, the benefits of providing an interdisciplinary experiential learning program at rehabilitation centres significantly impact several groups and can positively affect organizations as well. Understanding the various considerations that could further enhance or hinder the implementation of this program is key to optimizing the outcomes and providing better experiences and patient care. Overall, this study provided valuable insight into the potential enablers and barriers to developing an interdisciplinary experiential learning program for university students within rehabilitation centres. This initiative could further enhance educational curriculums, student and clinical experiences, patient outcomes, and organizational goals. Through this qualitative study and multi-perspective lens, the presented recommendations provide key areas to focus on while developing and maintaining experiential learning programming.

## Data Availability

The raw data supporting the conclusions of this article will be made available by the authors, without undue reservation.

## Appendix A

### Semi-structured interview guide

*What is your profession or role?*

(Rehabilitation centre leader, clinician, university faculty/staff, or student/alumni).

*How many years have you been in your role (years of experience)?*

*The rest of the questions are more specific to the implementation of a new interdisciplinary approach*.

1. Do you think there is a need to create an interdisciplinary approach to support the programs already established?
2. Do you think there are reasons why there is not already one? Please elaborate further. (Prompt—awareness of existing barriers, knowledge from other experiences)
  ○ Rehab leaders: Are there procedural or organizational barriers at (rehabilitation centre)?
  ○ Clinicians: Is there a lack of supervisors available to take on students? Is it not a preferred approach?
  ○ University faculty/staff: Would a placement like this be doable—would it fit within the curriculum?
  ○ Students/alumni: From your experience, have you gained valuable experience/knowledge?
3. What types of experience or skills should students have to increase the quality of this approach? (Prompt—previous student experience, level of education, personal interests)
  ○ Rehab leaders: Specific qualifications needed at (rehabilitation centre)?
  ○ Clinicians: How are students assigned a supervisor?
  ○ University faculty/staff: What was the process for students to take the experiential learning course before?
  ○ Students/alumni: Have previous experience?
4. Based on your past experience/placement, do you think the implementation of this approach is feasible? (Prompt—physical location, cost, support, skills, travelling/commuting)
  ○ Rehab leaders: Can placement for students work with staff and within (rehabilitation centre)? (Physical location, cost, hospital overseeing/supervision)
  ○ Clinicians: Do you think enough staff would agree to work with students? How about patients? (Staff support, patient agreement)
  ○ University faculty/staff: What things would need to be included to fit with the curriculum? (Educational policies)
  ○ Students/alumni: Could you see yourself being able to fully commit to this approach? (Travelling time, time management, motivation)
5. Would this approach align with your professional identity or with the organization's mission/vision?
  ○ Rehab leaders: (Rehabilitation centre)/hospital or specific program
  ○ Clinicians: Any health staff member in the program you work in
  ○ University faculty/staff: University or faculty or department
  ○ Students/alumni: Student, i.e., the program you are in or went through
6. How easy or difficult would it be for you or your organization to engage in this approach? (Prompt—time management, larger care team, more communication, independence, etc.)
  ○ Rehab leaders: (Rehabilitation centre)/hospital—policies, cost
  ○ Clinicians: Staff members—workload, patient care/care team, communication
  ○ University faculty/staff: University—policies, consistency, curriculum, cost
  ○ Students/alumni: Student—time management, workload, cost
7. How confident are you that this approach would be beneficial? (Prompt—pertaining to skills, patients, working/learning environment)
  ○ Rehab leaders: At (rehabilitation centre), help with other initiatives/programs
  ○ Clinicians: To student learning, work environment, cohesiveness/teamwork
  ○ University faculty/staff: Help with curriculum, easier to add similar approaches in the future
  ○ Students/alumni: Adding to your skills/experience, exposure to new environments/people
8. What outcomes do you think could occur after implementing an approach like this? (Prompt—are these outcomes negative or positive? Short-term or long-term?)
  ○ Rehab leaders: Adds complications at the hospital, navigating issues—other approaches can be implemented
  ○ Clinicians: Communication, workload, patient care
  ○ University faculty/staff: Fit with curriculum
  ○ Students/alumni: Skills or experience achieved or lacked
9. What do you think the goal of an approach like this should be? What would success look like?
  ○ Rehab leaders: More exposure to (rehabilitation centre), a successful approach to ensure projects are completed with more perspectives, and that can be incorporated into other programs
  ○ Clinicians: Help guide aspiring students, patients benefit
  ○ University faculty/staff: Beneficial learning experience
  ○ Students/alumni: Help with student experience, more exposure, working with a bigger team and other students
10. How should this approach be evaluated?
  ○ Rehab leaders: Could this approach help with the hospital's organization? What is the key metric you would use to evaluate the program?
  ○ Clinicians: What would you think should be measured to indicate if this is helping?
  ○ University faculty/staff: What is the key metric you would use to evaluate the program?
    Students/alumni: Based on your previous learning, would using a more interdisciplinary approach enhance your overall practicum experience and skills? What should be measured to determine if this has been helpful?
11. On a scale from 1 to 10, 10 being the most important, how important do you think it is to create an interdisciplinary approach to experiential learning? Can you elaborate on why?

## Appendix B

**Table B1:** Coding guidelines.

TDF Domain	Barriers	Enablers
Knowledge	• Lack of knowledge • Different kinds of knowledge/understanding	• Extra insight or knowledge • Consistent understanding across teams/organizations
Skills	• Lack of skill or training (inexperience)	• More practical experience
Environment	• Physical environment • Economic feasibility • Time management • Distance from location • Attitude differences	• Economic and organizational support • Physical location is feasible • Support from other members and patients
Professional identity	• Different organizational mission • Does not align with professional identity	• Complementary organizational missions • Helps with professional role/aspirations
Beliefs and capabilities	• Lack of belief in abilities (for each role) • Boundaries to one's capabilities (personal and organizational)	High confidence in being effectively involved with the program
Optimism	• Program won't aid (list specifics)	• Program will aid (list specifics)
Intentions	• Low belief in the importance of the program	• High belief in the importance of the program
Goals	• Lack of goals	• Stated goals that help students, faculty, and patients
Beliefs and consequences	• Negative outcomes • Worries and concerns	• Positive outcomes
Reinforcement	• No incentives	• Intrinsic motivations • Extrinsic motivations

## Appendix C

**Table C1:** Coverage of each TDF domain within each overarching theme.

TDF domain	Enabler *N* = 567	Barrier *N* = 207	Neutral/program development *N* = 360
Environment/resources	*n* = 301 (53%)	*n *= 165 (80%)	*n* = 155 (43%)
Reinforcement	*n* = 245 (43%)	20 (10%)	35 (10%)
Beliefs and consequences	*n* = 203 (36%)	14 (7%)	24 (7%)
Optimism	*n* = 184 (32%)	9 (4%)	0
Professional identity	*n* = 174 (31%)	26 (13%)	36 (10%)
Goals	*n* = 172 (30%)	15 (7%)	163 (45%)
Knowledge	*n* = 140 (25%)	35 (17%)	41 (11%)
Skills	*n* = 119 (21%)	17 (8%)	50 (14%)
Beliefs and capabilities	*n* = 78 (14%)	69 (33%)	42 (12%)

Quotes may be coded in more than one TDF domain and may have some overlap. Percentages will not equal to 100.
